# High-Flow Oscillatory Ventilation: A Possible Therapeutic Option for Pediatric Patients with Cardiovascular Diseases

**DOI:** 10.3390/pediatric16040079

**Published:** 2024-10-24

**Authors:** Stefano Scollo, Luigi La Via, Piero Pavone, Marco Piastra, Giorgio Conti, Carmelo Minardi

**Affiliations:** 1 Clinical Critical Care Fellow—PGY1, The Hospital for Sick Children, Toronto, ON M5G 1E8, Canada; stefanoscollo@hotmail.it; 2 Department of Anesthesia and Intensive Care 1, Azienda Ospedaliero Universitaria Policlinico, G. Rodolico—San Marco, 95123 Catania, Italy; minardi.carmelo@virgilio.it; 3 Clinical Pediatrics Unit, Department of Clinical and Experimental Medicine, University of Catania, 95123 Catania, Italy; piero.pavone@unict.it; 4 Institute of Anesthesia and Intensive Care, Catholic University of the Sacred Heart, 00168 Rome, Italy; marco.piastra@unicatt.it (M.P.); giorgio.conti@unicatt.it (G.C.)

**Keywords:** HFOV, pediatric critical care, congenital heart disease, acute respiratory distress syndrome, ARDS, pulmonary hypertension, mechanical ventilation, newborn

## Abstract

High-flow oscillatory ventilation (HFOV) is a common rescue treatment in infants and children with respiratory failure. This type of ventilation is an effective technique in numerous diseases that affect a child in the postnatal period, such as ARDS, meconium aspiration syndrome (MIS), postnatal pulmonary bleeding and idiopathic pulmonary hypertension (IPH). Although this ventilation technique is commonly recognized as a valuable therapeutic option in the general pediatric population, this is not the same for children with congenital cardiovascular diseases. The key mechanism of oscillatory ventilation is continuous positive pressure administered within the airways via a small tidal volume at high frequency. Tidal volumes are between 1 and 3 mL/kg delivered at 5–15 Hz, equivalent to 300–900 breaths per minute. A few older studies conducted on humans and animals highlight that HFOV may be dangerous for congenital heart patients. According to these evidences, hemodynamic parameters such as blood pressure, wedge pressure, central venous pressure, heart rate and inotrope level can be dangerously changed for patients with congenital heart disease; therefore, oscillatory ventilation should be avoided. Numerous retrospective studies have pointed out how oscillatory ventilation constitutes a valid therapeutic option in children with congenital heart disease. Recently, new evidences have highlighted how hemodynamic parameters are modified in a non-significant way by this type of ventilation, remaining beneficial as in the normal pediatric population. This narrative review aims to describe the mechanisms of oscillatory ventilation and collect all the available evidences to support its use in pediatric patients with congenital heart problems.

## 1. Introduction

High-frequency oscillatory ventilation (HFOV) is a lung-protective strategy used across different patient populations, from neonates to adults, particularly in cases of acute lung injury (ALI) or acute respiratory distress syndrome (ARDS). It is commonly employed when conventional ventilation fails to achieve adequate oxygenation or worsens lung injury [1]. In addition, HFOV has been used for neonatal respiratory support for over 30 years, delivering tidal volumes at or below dead space. Research shows HFV can improve lung function and reduce ventilator-induced lung injury, a key risk factor for bronchopulmonary dysplasia (BPD). Mastery of its mechanics, physiological effects and safe application is vital for optimal use in clinical practice [2]. Oscillatory ventilation may require prolonged sedation and neuromuscular blockade to be effective, resulting in prolonged ICU requirement [3,4,5,6,7]. Cardiovascular complications include reduced venous return, decreased cardiac output, intraventricular hemorrhage and increased intrathoracic pressure [8,9]. Pneumothorax, pneumomediastinum, pneumopericardium, and interstitial emphysema may also occur during HFOV [10,11]. Various clinical studies have found that its use is safe and beneficial even in pediatric patients with univentricular physiology, as in the case of those undergoing Fontan surgery [12]. This type of ventilation has been effectively used in the treatment of ARDS as an early-rescue ventilation after cardiac surgery, performed in cardiopulmonary bypass or in the treatment of post-operative pulmonary hypertension [13,14,15]. This narrative review aims to highlight how and why using this ventilation mode is a valuable aid in the treatment of clinical conditions that may occur after cardiopulmonary bypass and how it is a safe and beneficial ventilation technique in pediatric patients suffering from congenital heart disease.

## 2. HFOV: How It Works

**Figure 1:**
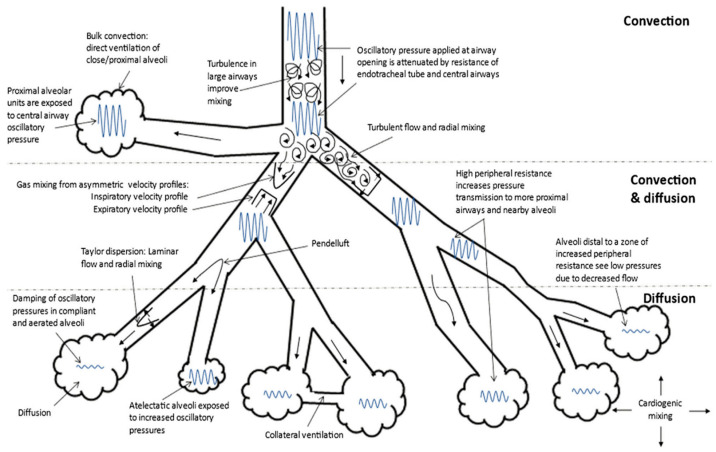
Gas transport mechanisms during high-frequency oscillatory ventilation (HFOV). Adapted from references: [17,23].

Oscillatory ventilation is a mode of invasive mechanical ventilation that requires the use of a particular ventilator called an oscillator [16]. The mechanism of oscillatory ventilation is based on the generation of an oscillatory flow inside the airways, creating a waveform that varies depending on the source of the oscillation, the attenuation of the waveform during the passage inside the airways and the pressure and efficiency of the volume delivered to the alveoli [17,18]. The HFOV then produces a biphasic pressure wave and diverts the new flow—called bias flow—to the patient with frequencies above 3 Hz [19]. The transmission frequencies of the oscillatory wave flow may vary from 5 to 15 Hz, equivalent to a range from 300 to 900 acts/min, through a small tidal volume which oscillates between 1 and 3 mL/kg [20,21]. Through lung recruitment, the use of small tidal volumes reduces the incidence of atelectrauma determined by the process of the opening and closing of the alveoli performed by conventional ventilation, reducing the incidence of both volotrauma and barotrauma contemporarily [20,21]. The positive and negative deflections of the flow created by the oscillation lead to a fusion between the inspiratory and expiratory phases, which occur simultaneously. During conventional mechanical ventilation, gas transfer occurs by mass transport of gas molecules from the large central airways to the smaller peripheral airways. This requires the tidal volume to be greater than dead space ventilation. During HFOV, this is not possible because the current ventilation delivered is less than the dead space [17,21,22]. Compared to conventional ventilation, the transport of gas in oscillatory ventilation occurs with a very different dynamic, which involves the participation of multiple mechanisms simultaneously [23]. A well-known mechanism for gas transfer in HFOV is convective mass transfer, which may contribute to gas exchange in the proximal airways even though it plays only a minor role in peripheral gas exchange [17,21,22]. Turbulence is another method of gas transfer, especially in larger airways. In this case, the different velocity profiles of the various asymmetric particles will lead to a net convective transport (Figure 1).

This mode of gas exchange is most often observed in the bifurcation of the airways [17,21,22]. Taylor’s dispersion and molecular diffusion are one of the most essential mechanisms of gas exchange during HFOV [17,23]. Other mechanisms described include the Pendelluft effect, cardiogenic mixing and collateral ventilation [17,23]. Oscillatory ventilation is a rescue therapy in multiple clinical conditions such as refractory hypoxemia, severe “air leak” syndromes and cardiovascular failure [24,25,26].

## 3. Indications and Contraindications

There are many indications, contraindications and risks in the use of HFOV, as shown in Table 1.

For instance, HFOV is advantageous for patients with severe respiratory failure, where conventional ventilation is set to parameters that can induce volotrauma, barotrauma and atelectrauma [3,5,17,27,28,29]. Oscillatory ventilation is advantageous in patients affected by ventilation-induced lung injury (VILI) and ARDS [27,28,29]. Among newborns, HFOV is indicated for patients with neonatal air leak syndrome, persistent pulmonary hypertension and meconium aspiration [20,30]. Even though there are no specific contraindications in the use of HFOV, it is mandatory to consider any possible side effects. The use of HFOV in preterm infants with pneumothorax is contentious. While some reports suggest that it may be a viable conservative treatment option, there is a lack of large-scale studies to support this practice [25]. Other experts advise against its use due to potential risks and complications [10,11]. Further research is needed to evaluate the safety and efficacy of HFOV in this context. Increased airway resistance, hyperinflation and air-trapping must be considered as a source of potential damage to the patient [8,9]. Therefore, aggressive ventilation parameters can determine lung hyperinflation and hesitate in barotrauma, pneumomediastinum, pneumopericardium and interstitial pulmonary emphysema as natural complications [10,11]. HFOV can reduce venous return and cardiac output, and cause intraventricular hemorrhage due to the increase in intrathoracic positive pressure, potentially worsening the hemodynamic status of the patient [8,9]. In this context, the ventilatory setting should be properly titrated in patients with congenital heart disease, focusing on the impact on venous return, cardiac output and increase in intrathoracic positive pressure. During oscillatory ventilation, prolonged intubation might be required, thus increasing the risk of infectious complications (such as pneumonia and sepsis) and length of stay in the ICU [10,31]. Furthermore, it is essential to consider that the transportation of the patient is impossible and, because of the noisy piston pump, carrying out the clinical examination to detect any complications might be very difficult [3]. Airway aspiration maneuvers could reveal changelings because of the strong alveolar de-recruitment. It is mandatory for the clinician to establish previously the methods and times based on the clinical condition of the patient [32]. The presence of plugs or mucous secretions could obstruct airways and hinder a correct thoracic oscillation, compelling the clinician to practice suctioning maneuvers [26,33]. The use of a closed circuit for aspiration does not prevent alveolar de-recruitment. To maintain optimal alveolar function after a procedure, the clinician should resume performing ventilation while incorporating post-procedural recruitment maneuvers. These maneuvers are crucial for reopening and keeping alveolar structures expanded, ensuring adequate gas exchange and preventing atelectasis [5,32,34]. The disconnection of the patient from the ventilator should only be performed if necessary [35].

## 4. Ventilator Settings

**Figure 2:**
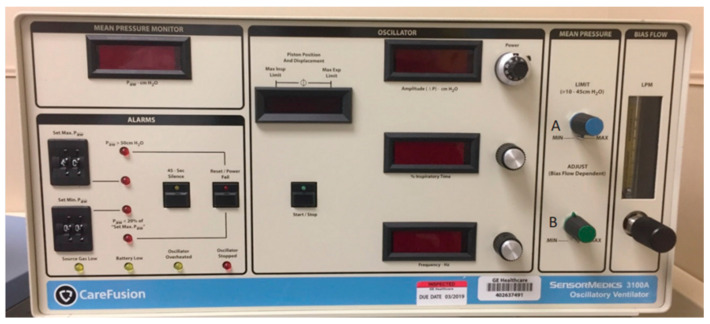
Example of a ventilator with HFOV modality.

Figure 2 shows an example of a ventilator that might be used for HFOV.

The most common technique for establishing initial ventilator settings is to start with a MAP between a range of 2–3 cm H_2_O above the mean airway pressure in conventional ventilation and a frequency between 10 and 12 Hz based on the age of the patient, leaving the other parameters stable. Therefore, after determining the MAP and rate, the approximal power setting is set between 70 and 90 cm H_2_O and the bias flow is typically 20 to 40 L/min with an inspiratory time of 33% [14]. There are no specific indications in the literature on the management of ventilatory settings, but the most common is the “open lung strategy”, which is based on the judicious titration of the MAP. The procedure consists of a progressive increase of 2 cm H_2_O on the MAP knob, evaluating the SpO_2_ every 2–3 min until there is no more gain in terms of peripheral saturation value. This pressure corresponds to “hyperinflation pressure”. Next, the knob is turned down by 2 cm H_2_O until the SpO_2_ decreases. The level of MAP obtained corresponds now to “de-recruitment pressure”. Subsequently, the knob is raised by 2 cm H_2_O above the “de-recruitment pressure”, obtaining the optimal MAP [36]. It is mandatory to check that the lungs are not hyperinflated with a chest X-ray or ultrasound: the lower edge must not exceed the ninth rib [37]. Furthermore, it is essential for the clinician to evaluate the stability of the patient’s hemodynamic conditions. The other parameters must be adjusted, reaching an SpO_2_ value of at least between 88 and 92% with a pH ≥ 7.20 tolerating permissive hypercapnia [14].

## 5. HFOV in Newborns and Children with Heart Disease

The main doubts regarding the use of HFOV concern the possible hemodynamic impact that this mode ventilation may have on children with congenital heart disease. However, there is no evidence in the literature to prove that this ventilation mode can be used in the same conditions that can occur in patients not affected by congenital pathologies [28,38,39]. Numerous clinical studies have demonstrated that the perception of the hemodynamic impact of HFOV is often overrated, both in normal and congenital heart disease populations. The actual impact on hemodynamics is frequently less significant than initially perceived [40,41,42,43,44,45]. Two clinical studies conducted on a pediatric population showed how cardiac output was reduced when switching from conventional ventilation to HFOV in patients with ARDS, but they were very small single-center studies, composed of small preterm populations, and in one of them, the blood pressure was measured noninvasively [46,47]. Fort et al., in a study on an adult population, found an increase in pulmonary wedge pressure without significant alterations on other hemodynamic parameters such as cardiac output [48]. Clinical studies in adult populations have demonstrated that when switching from conventional ventilation to oscillatory ventilation in patients with ARDS, there is a significant increase in pulmonary wedge pressure and central venous pressure, without changes in heart rate, blood pressure and cardiac output [28,38]. Similar results have been found in studies on pediatric populations [44,45]. It was seen by Gutiérrez et al. in a single patient that a possible reduction in cardiac output and cardiac index may occur in the recruitment phase, indicating possible alveolar overdistention; in contrast, Goodman et al. observed an improvement in hemodynamic parameters such as cardiac index, cardiac output and oxygen transport accompanied only by a slight reduction in heart rate. This possible effect was explained by two contemporary mechanisms: the reduction in paCO_2_ and the reduction in transthoracic impedance. The correction of hypercapnia-induced acidosis and reduction in cardiac afterload improved patients’ cardiovascular performance on both fronts [44,45]. While clinical studies on the hemodynamic effects of oscillatory ventilation in the pediatric population are rare, those on the pediatric population affected by congenital or acquired heart disease are even rarer. De Jager et al. recently conducted a retrospective analysis on 52 patients suffering from congenital and/or acquired heart disease with respiratory failure: no significant changes in hemodynamic parameters such as heart rate, blood pressure, central venous pressure and lactate level were measured. No significant changes were detected in the number of fluid boluses made when switching from one ventilatory mode to another, nor significant alterations in the administration of vasoactive drugs. Furthermore, an improvement in respiratory parameters such as paCO_2_ and paO_2_ was detected, associated with a simultaneous significant reduction in the dosage of muscle relaxants without any significant change in the dosage of sedatives. This monocentric and retrospective study is the only one in the literature that aims to analyze the hemodynamic effects of HFOV in this peculiar population of pediatric patients. It should also be underlined that the inclusion criteria were established not by considering clinical parameters, but by considering ventilatory parameters. The transition from the conventional to the oscillatory ventilation mode was carried out by adopting a protocol that was determined by the achievement of harmful ventilatory parameters, this was a cut off to use the HFOV regardless of the clinical condition and the underlying cardiac pathology. In this way, it was possible to observe the effects of this ventilation mode on patients suffering from cardiac pathologies, including acquired or congenital ones [14]. HFOV has been used profitably in the treatment of post-cardiopulmonary bypass ARDS for more than a decade now [13,15,40,43,49,50,51]. Oscillatory ventilation has been shown to improve oxygen index, PaO_2_/FiO_2_ ratio, blood alveolar–arterial oxygen concentration gap, dynamic lung compliance, PaO_2_, PaCO_2_ and pH, resulting in a positive impact on patient survival [15,43,51,52]. HFOV proved to be beneficial in controlling the production of inflammatory mediators during ARDS. Zheng et al. demonstrated that the use of oscillatory ventilation with protective parameters mixed with the “volume guaranteed” mode led to a significant improvement in the panel of proinflammatory mediators such as IL-6, IL-8 and TNF-α that are produced during the inflammatory response in ARDS. The reduction in these activators significantly improved the blood gas analysis parameters with a decrease in days of post-operative mechanical ventilation [53]. Oscillatory ventilation also represents a valuable aid in the treatment of pulmonary hypertension in cardiac newborns. The use of HFOV and inhaled nitric oxide therapy is now a consolidated therapeutic option for neonatal pulmonary hypertension. Its use is also finding its place in the pediatric population affected by congenital heart disease [42,53]. Huang et al. have demonstrated that, as in patients suffering from cardiac congenital disease, oscillatory ventilation associated with nitric oxide is a concrete and safe therapeutic option in the treatment of post-cardiac surgery pulmonary hypertension, improving blood gas analysis parameters while determining a significant reduction in the days of post-operative mechanical ventilation [42].

## 6. Conclusions

Oscillatory ventilation plays a role as a clinical option in pediatric patients suffering from congenital heart disease. HFOV has potential as a rescue therapy for pediatric patients with congenital cardiac conditions who are affected by acute respiratory distress syndrome (ARDS). Retrospective studies suggest that HFOV could also serve as an early protective ventilation strategy in this particularly vulnerable population. Given that many congenital cardiac patients are often born preterm, HFOV should not be dismissed as a viable option for protective ventilation in these fragile patients. Moreover, due to the risk of hemodynamic side effects associated with HFOV, it is essential to implement thorough hemodynamic monitoring. Techniques such as invasive blood pressure measurement and regular functional echocardiography can provide valuable insights for titrating HFOV settings effectively, ensuring that both respiratory support and hemodynamic stability are maintained. So far, there are no double-blind randomized controlled trials that prove its superiority over conventional ventilation in the clinical conditions for which it is indicated. Retrospective analyses have shown that it can also have a beneficial role in the treatment of pathologies such as ARDS and pulmonary hypertension in the pediatric population affected by congenital heart diseases. Given the rarity of this type of population, as well as the prejudice around this ventilatory modality, its diffusion is still limited. However, although rare and limited by the small size of the samples and by the prevalence of single-center retrospective studies, all publications for more than a decade have highlighted the benefit of HFOV in the pediatric population affected by congenital heart disease.

## 7. Future Directions

The future of HFOV in pediatric patients, particularly those with congenital heart disease, hinges on further rigorous research and refinement of clinical practices. While existing retrospective studies indicate that HFOV can be beneficial, there is a critical need for well-designed, multicentric, randomized controlled trials to establish robust evidence of its efficacy and safety compared to conventional ventilation. Research should focus on optimizing HFOV settings to minimize potential hemodynamic impacts and complications such as barotrauma and pneumothorax. Additionally, exploring the synergistic effects of combining HFOV with other therapeutic modalities, such as inhaled nitric oxide, could provide new avenues for enhancing patient outcomes. Future studies should also aim to develop tailored protocols that account for the unique physiological and pathological conditions in pediatric patients with congenital heart disease. Furthermore, advancements in technology and ventilator design could help mitigate some of the current limitations associated with HFOV, such as difficulties in clinical examination and airway management. By addressing these research gaps, we can better define the role of HFOV in treating severe respiratory conditions in this vulnerable patient population and potentially broaden its application in critical care settings.

## Figures and Tables

**Table 1:** Indications and contraindications of HFOV.

Indications	Contraindications
Pulmonary issues	Pulmonary/thoracic complications
ARDS. Ventilator-associated lung injury. Alveolar hemorrhage. Large air leak with inability to keep the lungs open. Failure of conventional mechanical ventilation. Refractory hypoxemia. Pulmonary interstitial emphysema. Meconium aspiration. Pulmonary hypoplasia. Bronchopulmonary fistulae.	High intrathoracic pressures. Pneumothorax. Bronchospasm. Airway obstruction. Barotrauma. Pneumomediastinum. Subcutaneous emphysema. ET migration/displacement.
Cardiovascular diseases	Cardiovascular contraindications
Persistent pulmonary hypertension.	High right ventricular preload. Increased pulmonary capillary wedge pressure.
Possible indications	Other contraindication
Abdominal compartment syndrome. Increased intracranial pressure.	Multiple organ failure. Sepsis. Refractory acidosis. Intraventricular hemorrhage. Cellular injury.

ARDS: acute respiratory distress syndrome; ET: endotracheal tube.

## Data Availability

No new data were created for this study.
